# A randomized trial comparing the intraoperative durability of double-gloving with Biogel® surgical gloves to 3 comparators

**DOI:** 10.1017/ash.2024.431

**Published:** 2024-10-10

**Authors:** Michelle Doll, Asmaa Namoos, Le Kang, Jibanananda Satpathy, Michael J. Feldman, Anthony Cassano, Jaime Bohl, Michel B. Aboutanos, Brian Cameron, Jason Kim, Celine Asbury, Mahdee Haque, Olivia Hess, Henrik Ahlbom, Gonzalo Bearman

**Affiliations:** 1 Virginia Commonwealth University School of Medicine, Richmond, VA, USA; 2 Molnlycke Healthcare, Gothenburg, Sweden

## Abstract

**Objective:**

To determine and compare the intraoperative durability of 4 major surgical glove brands.

**Design, Setting, and Participants:**

This study is a randomized open-label clinical trial in which surgical gloves from 4 manufacturers are randomized to 5 surgical subspecialty study groups: (1) orthopedic surgery, (2) neurosurgery, (3) colorectal surgery, (4) trauma or acute general surgery, and (5) cardiac and plastic surgeries. The study was divided into 10 periods, with a cross-over design, and was conducted at a tertiary care academic medical center. Participants were licensed and certified physicians, physicians-in-training, scrub nurses, or technicians working within the sterile field.

**Interventions:**

Participants from each study group were randomly assigned to 1 of 4 surgical glove manufacturer types and subsequently rotated through the other 3 glove brands such that each participant acted as their own control in the sequential cross-over design.

**Main Outcomes and Measures:**

The primary outcome was to determine and compare the intraoperative failure rate of Biogel® Sterile Surgical undergloves against sterile surgical undergloves from 3 other manufacturers, both as a combined competitor group and individually.

**Results:**

There were no differences between brands with respect to the primary outcome of underglove intraoperative failures. Brand 1 wearers were slightly more likely to detect glove failures when they occurred.

**Conclusion:**

The durability of surgical gloves intraoperatively is similar across 4 major glove manufacturers. Detection of intraoperative failures is infrequent, though specific glove characteristics may promote enhanced detection. Recognition of glove perforations intraoperatively is important in the maintenance of a maximally sterile field.

**Trial Registration:**

ClinicalTrials.gov Identifier: NCT03344354.

## Introduction

Sterile surgical gloves serve a critical barrier role at the interface between healthcare providers and patients during invasive procedures. Surgical glove integrity at this interface has been the subject of intense study for decades.^1^ Perforation rates are well documented, albeit highly variable,^1,2^ depending on factors such as type of surgery,^3–5^ duration of surgery,^5–8^ role of the glove wearer,^4,7^ latex content,^1,6^ and thickness of gloves,^2,5^ manufacturer,^6^ and method of perforation detection.^1,2^ The implications of these perforations remain a subject of debate,^9^ as it is difficult to attribute surgical site infections (SSIs) to a specific source. Nevertheless, there are multiple examples of SSI outbreaks with epidemiologic linkage to specific organisms in the hands of a surgical team member.^10^ In an era of increased scrutiny of SSI rates, the efficacy of this critical barrier is a primary concern for surgical stakeholders.

Surgical gloves have evolved since many of the existing studies of glove integrity were performed. Non-latex gloves make up the majority of gloves in use at surgical centers in the United States and an increasing proportion internationally as well.^11,12^ In addition, double-gloving is now a standard in operating rooms in the United States as this practice is associated with significantly decreased rates of inner glove perforation without compromising glove functionality.^2,5^ It is important to note that the majority of perforations of surgical gloves during procedures are not detected by the glove wearer.^5,7,13^ Indicator glove systems, where the inner glove is a striking color contrast to the outer glove, increase wearer detection of glove perforations, allowing providers the opportunity to change gloves when breaches in the outer barrier occur.^2,14^

Standardized surgical glove integrity studies are limited in the current medical literature. Typically, gloves under study are the default products in use in a clinical setting or a small supply of intentionally chosen gloves for investigation in a laboratory setting. Variability in gloves by style and manufacturer limits the generalizability of these strategies.^6^ Clearly, new studies to understand glove perforation risks in the modern surgical era would be valuable.

We conducted an open-label randomized clinical trial using products from 4 major glove manufacturers to better define and compare surgical glove integrity during major operative procedures. The full protocol can be accessed as Supplement 1.

The **primary objective** of the investigation is to determine and compare the intraoperative failure rate of Biogel® (Brand 1) surgical undergloves against undergloves from 3 other manufacturers, both as a combined competitor group and individually.

The **secondary objectives** include:

- Comparison of failure rates of overgloves of Brand 1 with those of 3 other manufacturers, both as a combined competitor group and individually.
- Determination and comparison of perforation rates of undergloves and overgloves of Brand 1 with those of 3 other manufacturers.
- Determination of the frequency of glove wearer detection of overglove perforation when wearing Brand 1 Indicator® System and comparison to that of 3 other manufacturers.
- Evaluation of glove wearer perceptions of fit, comfort, tactile sensitivity, and hand fatigue for Brand 1 surgical gloves and compared with those of 3 other manufacturers.

## Methodology

### Study design

This study, divided into 10 periods, is a cross-over randomized open-label clinical trial (Table 1) in which surgical gloves from 4 manufacturers were randomized in a 2:1 ratio (Brand 1 to each other brand) in each study period to 5 surgical subspecialty study groups: (1) orthopedic surgery, (2) neurosurgery, (3) colorectal surgery, (4) trauma/acute general surgery (AGS), and (5) cardiac and plastic surgeries. The one-time randomization of each subspecialty to a study group occurred prior to the onset of period 1, via a lottery method, where the first subspecialty drawn became group 1 (completed by MD). The glove assignments and rotation for each group were defined a priori as shown in Table 1 (by GB and HA). The study site is an academic, tertiary care center. Each period of the study was defined by a minimum number of gloves collected across all 5 groups, reflecting 320 targeted individual underglove gloving events. Gloves under investigation in this study include both latex and synthetic products. Each participant underwent a glove fitting, choosing preferred gloves from a variety of glove types for each brand. All gloves under study were commercially available and FDA approved for use in the United States. The study was approved by the Western Institutional Review Board. We followed the Consolidated Standards of Reporting Trials (CONSORT) reporting guidelines.

**Table 1.** Study randomization and cross-over

Period	Neurosurgery	Colorectal surgery	Orthopedic surgery	Cardiothoracic and plastic surgery	Trauma and acute general surgery	Total number individual undergloves/overgloves targeted per period:
1	Brand 1	Brand 4	Brand 1	Brand 3	Brand 2	320/540
2	Brand 2	Brand 1	Brand 4	Brand 1	Brand 3	320/540
3	Brand 3	Brand 2	Brand 1	Brand 4	Brand 1	320/540
4	Brand 1	Brand 3	Brand 2	Brand 1	Brand 4	320/540
5	Brand 4	Brand 1	Brand 3	Brand 2	Brand 1	320/540
6	Brand 1	Brand 4	Brand 1	Brand 3	Brand 2	320/540
7	Brand 2	Brand 1	Brand 4	Brand 1	Brand 3	320/540
8	Brand 3	Brand 2	Brand 1	Brand 4	Brand 1	320/540
9	Brand 1	Brand 3	Brand 2	Brand 1	Brand 4	320/540
10	Brand 4	Brand 1	Brand 3	Brand 2	Brand 1	320/540
Total Gloves:						3200/6400

*Each period ends when 1 brand has reached 160 individual underglove collections.

### Outcome variables

The overall failure of the gloves was assessed at 4 time points: (1) pre-donning failure (defect noted upon removal from packaging), (2) donning failure (failure of the glove while putting it on), (3) observed intraoperative failure (the provider notes a defect in glove integrity during a procedure), and (4) post-procedure, or overall failure, determined by water leak testing (WLT).

Perforation rates were defined as the number of holes/tears/defects per glove as a continuous count variable.

### Inclusion and exclusion criteria for participants

The inclusion criteria for study participants were (1) licensed physicians, physicians-in-training, scrub nurses, or technicians working in targeted surgery specialties, (2) active participants within the sterile field, (3) willing to wear a half-size larger underglove compared with overglove when recommended by manufacturer, and (4) willing to use latex gloves. Participants were enrolled by the study team, and they signed informed consent.

The exclusion criteria for study participants were (1) dermatological or other medical conditions that may prevent proper scrub technique, (2) wearing of hand jewelry during surgical procedures, (3) existing conflicts of interest with a glove manufacturer, and (4) latex allergy or objection to using latex gloves.

### Case selection

Procedure inclusion criteria were (1) at least 1 participating study clinician is scrubbing into the case and (2) the case expected duration is at least 1 hour as per surgical scheduling data. Cases involving latex-sensitive patients are excluded from the latex arms of the study. No patient information was collected.

### Study procedures

Participant gloves were distributed by the study team at the start of each case and collected by the study team in biohazard bags after use in the procedure.

### Glove testing

All study gloves used in a surgical procedure by participants were tested using a WLT standard procedure: D5151-19: Standard Test Method for Detection of Holes in Medical Gloves (ASTM International, Conshohocken, PA).^15^ The WLT machine was manufactured by DipTech Systems, Inc., Kent OH. Briefly, gloves suspended and filled with 1 liter water were inspected immediately and at 2 minutes for droplets. Any glove demonstrating defects was recorded as a failure, and the total number and site of holes/tears/punctures were recorded.

Laboratory technicians were trained on the machine and demonstrated proficiency.

### Provider participant questionnaires

Providers were asked to complete a questionnaire at the end of each period. Gloves were rated by a visual analog scale (VAS) on the following characteristics: tactile sensitivity, grip, comfort, fit, strength of glove, and effect on the skin (irritation). Characteristics were scored by measuring the distance from 0 to the participant’s mark to the nearest millimeter on a 0–100 mm scale with 0 mm being “very bad” and 100 mm being “very good.”

### Statistical analysis plan

The failure rate and all dichotomous outcome variables were analyzed with a two-sided Fisher’s exact test between the Brand 1 gloves and other brands. The difference in proportion and estimated odds ratio (OR) with their 95% confidence intervals (CIs) were calculated using the exact Fisher’s method. Mean perforation rates and their standard deviations were estimated between Brand 1 gloves and each of the other gloves and combined.

Generalized linear models were used in the analyses of failure rates, adjusting for other covariates, with binomial distribution and logit link function for the dichotomous outcomes specifically, and Poisson distribution and log link function for the continuous count outcomes, if applicable. Adjusted ORs with 95% CIs were provided from these analyses.

All VAS measurements were regarded as continuous variables. For comparison between Brand 1 gloves and all other gloves combined, nonparametric Wilcoxon rank sum testing was used, and the Kruskal–Wallis rank sum test was used for comparison between each individual brand.

All tests were two-tailed and conducted at a 0.05 significance level unless otherwise specified. All analyses were performed using SAS® v9.4 (SAS Institute, Cary, NC, USA) and R Statistical Software, v4.3.0 (R Foundation for Statistical Computing, Vienna, Austria).

### Sample size determination

In order to detect a difference in the failure rate of undergloves between Brand 1 gloves and any one of the other manufacturer gloves with two-sided Fisher’s exact test at significance level 0.05 with a power of 80%, a total of 1,650 gloves were needed assuming a failure rate of 1% in Brand 1 gloves and 3% in the comparators combined.^2^ Assuming a dropout rate of 10%, a total of 1,833 undergloves were calculated to be needed. The same power for overgloves implies 1,833 total overgloves required.

## Results

Seventy-nine unique surgical team members participated in the study. Surgeons made up 44% (n = 35), surgical technicians 54% (n = 43), and 1 physician assistant participated (1%). The primary service of the participants was orthopedic surgery for 22 (28%), neurosurgery for 14 (18%), colorectal surgery for 19 (24%), AGS for 13 (16%), and cardiothoracic/plastic surgery for 11 (14%). The study ran from May 2018 through November 2021. The study was paused from March to June 2020 due to the coronavirus disease 2019 pandemic.

**Figure 1.**
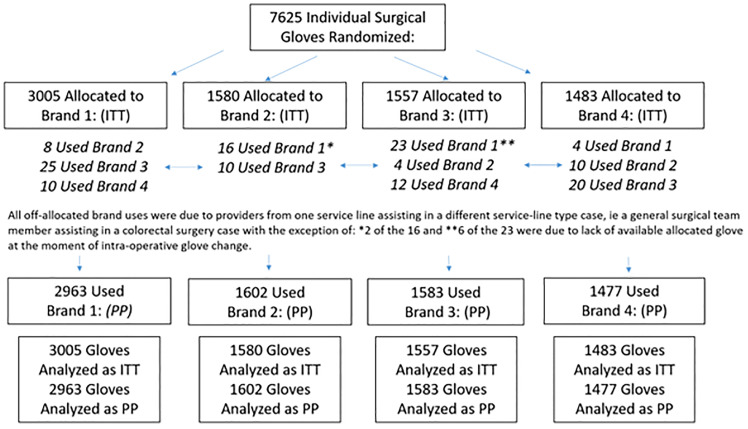
CONSORT flow diagram.

The total number of gloves collected within each brand and the primary and secondary outcomes are shown in Table 2 (intention to treat, ITT) and 3 (per protocol, PP). Overall, 7,625 gloves were analyzed (Figure 1), providing sufficient statistical power for comparisons between Brand 1 and each of the comparators and combined.

**Table 2.** Primary and secondary endpoints, intention to treat

Undergloves	Brand 1 n = 1408 (reference)	Brand 2 n = 713	Brand 3 n = 682	Brand 4 n = 704	Brands 2,3,4 Combined n = 2099
Gloves with any perforation (failure) (n (%), *P* value)	156 (11.1%)	87 (12.2%), *P* = .471	70 (10.3%), *P* = .600	54 (7.7%), *P* = .014	211 (10.1%), *P* = .339
Number of perforations (mean (SD), *P* value)	0.143 (0.459)	0.174 (0.531), *P* = .353	0.142 (0.480), *P* = .383	0.122 (0.535), *P* = .025	0.146 (0.517), *P* = .295
Intraoperative failure (detected by operative team) (n (%), *P* value)	54 (3.8%)	14 (2.0%), *P* = .023	38 (5.6%), *P* = .076	21 (3.0%), *P* = .278	73 (3.5%), *P* = .044
Donning failure (n (%), *P* value)	5 (0.4%)	0 (0%), *P* = .113	2 (0.3%), *P* = .528	2 (0.3%), *P* = .468	4 (0.2%), *P* = .032
Doffing failure (n (%), *P* value)	6 (0.4%)	0 (0%), *P* = .081	2 (0.3%), *P* = .490	6 (0.9%), *P* = .216	8 (0.4%), *P* = .065
**Overgloves**	Brand 1 N = 1597 (reference)	Brand 2 N = 867	Brand 3 N = 875	Brand 4 N = 779	Brands 2,3,4 Combined N = 2521
Gloves with any perforation (n (%), *P* value)	280 (17.5%)	175 (20.2%), *P* = .115	207 (23.7%), *P* < .001	140 (18.0%), *P* = .819	522 (20.7%), *P* = .012
Number of perforations (mean (SD), *P* value)	0.267 (0.709)	0.360 (0.925), *P* = .146	0.374 (0.839), *P* = .017	0.288 (0.782), *P* = .634	0.342 (0.853), *P* = .169
Intraoperative failure (detected by operative team) (n (%), *P* value)	90 (5.6%)	20 (2.3%), *P* < .001	57 (6.5%), *P* = .311	27 (3.5%), *P* = .027	104 (4.1%), *P* = .003
Donning failure (n (%), *P* value)	6 (0.4%)	0 (0%), *P* = .070	3 (0.3%), *P* = .509	2 (0.3%), *P* = .491	5 (0.2%), *P* = .022
Doffing failure (n (%), *P* value)	8 (0.5%)	0 (0%), *P* = .034	2 (0.2%), *P* = .345	7 (0.9%), *P* = .251	9 (0.4%), *P* = .030

### Undergloves

The primary outcome of the failure rate for undergloves between Brand 1 and all other brands combined was similar in both ITT and PP analyses: ITT: 11.1% versus 10.1%, *P* = .339 and PP: 11.0% versus 10.1%, *P* = .398. Comparisons between Brand 1 and each individual brand revealed no significant difference in failure rates, except between Brand 1 and Brand 4: ITT: 11.1% versus 7.7%, *P* = .014 and PP: 11.0% versus 7.7%, *P* = .016.

The secondary outcome of perforation counts per underglove is shown as a mean per glove in Tables 2 and 3. The rates of perforation (counts per glove) were no different except for the comparison Brand 1 with Brand 4 in both analyses in which there was a lower perforation count for Brand 4: ITT: Brand 1 rate 0.143 (SD 0.459) versus Brand 4 rate 0.122 (SD 0.535), *P* = .025 PP: Brand 1 rate 0.142 (SD 0.458) versus Brand 4 rate 0.123 (SD 0.537), *P* = .032 (Tables 2 and 3).

**Table 3.** Primary and secondary endpoints, per protocol

Undergloves	Brand 1 n = 1388 (reference)	Brand 2 n = 723	Brand 3 n = 696	Brand 4 n = 700	Brands 2,3,4 Combined n = 2119
Gloves with any perforation (Failure) (n (%), *P* value)	153 (11.0%)	87 (12.0%), *P* = .516	73 (10.5%), *P* = .765	54 (7.7%), *P* = .016	214 (10.1%), *P* = .398
Number of perforations (mean (SD), *P* value	0.142 (0.458)	0.172 (0.527), *P* = .363	0.145 (0.483), *P* = .470	0.123 (.537), *P* = .032	0.147 (0.517), *P* = .337
Intraoperative failure (detected by operative team) (n (%), *P* value)	54 (3.9%)	14 (1.9%), *P* = .016	38 (5.5%), *P* = .097	21 (3.0%), *P* = .267	73 (3.4%), *P* = .036
Donning failure (n (%), *P* value)	5 (0.4%)	0 (0%), *P* = .117	2 (0.3%), *P* = .468	2 (0.3%), *P* = .469	4 (0.2%), *P* = .028
Doffing failure (n (%), *P* value)	6 (0.4%)	0 (0%), *P* = .066	2 (0.3%), *P* = .439	6 (0.9%), *P* = .217	8 (0.4%), *P* = .048
**Overgloves**	Brand 1 n = 1575 (reference)	Brand 2 n = 879	Brand 3 n = 887	Brand 4 n = 777	Brands 2,3,4 Combined n = 2543
Gloves with any perforation (Failure) (n (%), *P* value)	280 (17.8%)	174 (19.8%), *P* = .233	209 (23.6%), *P* < .001	139 (17.9%), *P* = .954	522 (20.5%), *P* = .032
Number of perforations (mean (SD), *P* value)	0.269 (0.710)	0.353 (0.918), *P* = .205	0.374 (0.840), *P* = .021	0.287 (0.782), *P* = .611	0.340 (0.852), *P* = .213
Intraoperative failure (detected by operative team) (n (%), *P* value)	92 (5.8%)	20 (2.3%), *P* < .001	55 (6.2%), *P* = .406	27 (3.5%), *P* = .015	102 (4.0%), *P* = .001
Donning failure (n (%), *P* value)	6 (0.4%)	0 (0%), *P* = .061	3 (0.3%), *P* = .462	2 (0.3%), *P* = .491	5 (0.2%), *P* = .021
Doffing failure (n (%), *P* value)	8 (0.5%)	0 (0%), *P* = .036	2 (0.2%), *P* = .234	7 (0.9%), *P* = .254	9 (0.4%), *P* = .029

### Overgloves

Secondary endpoints in the ITT analysis revealed overglove (OG) failure rates that were lower for Brand 1 compared with all other brands and to each individual brand except for Brand 4. The OG failure rate was lower for Brand 1 compared with all other brands combined: 17.5% versus 20.7%, *P* = .012. Brand 1 compared with each individual brand as follows: Brand 2 failure rate was 20.2%, *P* = .115; Brand 3 was 23.7%, *P* < .001; and Brand 4 was 18.0%, *P* = .819 (Table 2). Similar results for the per protocol analysis were observed: Brand 1 OG failure rate was 17.8% versus all other brands 20.5%, *P* = .032. This overall difference was driven by the higher failure rate of Brand 3 at 23.6% (*P* = .001); Brands 2 and 4 were similar to Brand 1 in per protocol analysis (Table 3).

The secondary outcome of perforation counts per overglove is shown as a mean per glove in Tables 2 and 3. The only difference in brand-wise comparisons was that Brand 3 had a slightly increased perforation rate (count per glove) compared with Brand 1: ITT: 0.374 (SD 0.839) versus 0.267 (SD 0.709), *P* = .017, PP: 0.374 (SD 0.840) versus 0.269 (SD 0.710), *P* = .021, Tables 2 and 3.

### Intraoperative failures

Only a subset of perforations were detected by the glove wearer intraoperatively (Tables 2 and 3). Brand 1 intraoperative failures were more often detected than non-Brand 1 failures for undergloves (ITT: *P* = .044; PP: *P* = .036) and overgloves (ITT: *P* = .003; PP: *P* = .001). Each brand experienced a higher rate of intraoperative failures, or detection of breaches by the wearer, for overgloves compared with undergloves (Tables 2 and 3).

### Donning and doffing failures

Few donning or doffing failures occurred during the study (Tables 2 and 3). There was no difference in comparing these failure rates between individual brands except in the cases where 1 of the brands in pair-wise comparison had no failures observed during the course of the study. Brand 1 versus non-Brand 1 comparisons were significantly different statistically but driven by differences of Brand 1 versus Brand 2 gloves.

### Adjusted analysis

The overall rate of perforation was not different between Brand 1 and Brands 2,3,4 combined when controlling for surgical subspecialty group, case duration, side (R or L), and type of glove (underglove or overglove); the OR for combined brands was 1.061 (95% CI, 0.931–1.211) in reference to Brand 1 (Table 4).

**Table 4.** Adjusted analysis

	Crude		Adjusted	
Perforation event rate	OR	95% CI	OR	95% CI
Right side *(ref = left)*	0.792	0.699, 0.897	0.784	0.690, 0.891
Underglove *(ref = overglove)*	0.483	0.423, 0.551	0.490	0.428, 0.560
Surgical specialty *(ref = cardiothoracic surgery/plastic surgery)* Orthopedic surgery Colorectal surgery Neurosurgery Trauma/acute general surgery	2.004 1.134 0.803 1.308	1.687, 2.388 0.907, 1.414 0.611, 1.046 1.053, 1.623	2.163 1.069 0.810 1.344	1.811, 2.592 0.852, 1.338 0.616, 1.058 1.079, 1.674
Duration of procedure (min)	1.001	1.000, 1.001	1.002	1.001, 1.002
Brands 2,3,4 combined *(ref = Brand 1)*	1.111	0.978, 1.264	1.061	0.931, 1.211

Several variables affected the perforation rate. Gloves worn on the right hand were less likely to perforate (aOR = 0.78; 95% CI, 0.690–0.891) compared with the ones on the left hand, and undergloves were less likely to perforate than overgloves (aOR = 0.490; 95% CI, 0.428–0.560). In comparison to cardiothoracic/plastic surgery types as a reference, orthopedic and trauma/acute general surgeries experience a significantly higher perforation rate (aOR = 2.163; 95% CI, 1.811–2.592 and aOR = 1.344; 95% CI, 1.079–1.674, respectively), while colorectal and neurosurgery groups had similar perforation rates to the reference group. Increasing the duration of the procedure in minutes was associated with a small increase in both overall perforation rates (aOR = 1.002; 95% CI, 1.001–1.002) and intraoperative failures (aOR = 1.003; 95% CI, 1.002–1.003).

Observed intraoperative failures were less likely to be detected between combined Brands 2,3,4 versus Brand 1 (aOR = 0.733; 95% CI, 0.585–0.921) (Table 5). Similar to the overall perforation rate, undergloves were less likely to have observed failures compared with overgloves (aOR = 0.764; 95% CI, 0.606–0.960). There was no difference in observed intraoperative failures related to the hand side or between surgical groups with the exception of colorectal surgery for which providers were less likely to detect intraoperative failures (aOR = 0.350; 95% CI, 0.216–0.548) versus cardiothoracic/plastic surgery.

**Table 5.** Adjusted analysis

	Crude		Adjusted	
Intraoperative failures	OR	95% CI	OR	95% CI
Right side *(ref = left)*	0.943	0.754, 1.179	0.943	0.753, 1.181
Underglove *(ref = overglove)*	0.760	0.604, 0.954	0.764	0.606, 0.960
Surgical specialty *(ref = cardiothoracic surgery/plastic surgery)* Orthopedic surgery Colorectal surgery Neurosurgery Trauma/acute general surgery	0.890 0.400 0.698 1.205	0.668, 1.190 0.247, 0.622 0.450, 1.054 0.868, 1.669	1.035 0.350 0.716 1.299	0.769, 1.398 0.216, 0.548 0.461, 1.084 0.932, 1.806
Duration of procedure (min)	1.002	1.001, 1.003	1.003	1.002, 1.003
Brands 2,3,4 combined *(ref = Brand 1)*	0.789	0.631, 0.989	0.733	0.585, 0.921

### Participant questionnaires

Providers completed a total of 159 questionnaires over the 10 periods of the study: 45 (28%) from cardiothoracic/plastic surgery, 44 (28%) from orthopedic surgery, 24 (15%) from colorectal surgery, 27 (17%) from neurosurgery, and 19 (12%) from acute general/trauma surgery. The majority of questionnaires were completed by surgeons (n = 99, 63%), followed by OR scrub technicians (n = 51, 32%), and nurse procedure assistants (n = 8, 5%).

Mean satisfaction scores for each glove feature by brand are shown in Table 6. The differences in mean score between individual brands were not significant for most features: fit *P* = .137, comfort *P* = .195, grip *P* = .232, tactile sensitivity *P* = .330, and skin irritation *P* = .355. For the feature strength, participants reported higher satisfaction rankings for Brand 1 (*P* = .004). In comparisons of Brand 1 versus all other brands combined, multiple features were more highly ranked for Brand 1 including fit, comfort, grip, and strength (Table 6, far right column).

**Table 6.** Provider satisfaction scores^*^ for key glove features by brand

	Brand 1 Mean scores (SD)	Brand 2 Mean scores (SD)	Brand 3 Mean scores (SD)	Brand 4 Mean scores (SD)	Brand 2,3,4 Mean scores (SD)	*P* value Brand 1 vs Brand 2 vs Brand 3 vs Brand 4	*P* value Brand 1 vs Brand 2,3,4 combined
Fit	67.7 (26.2)	55.4 (30.6)	59.8 (29.7)	55.3 (33.6)	56.8 (31.1)	0.137	0.025
Comfort	66.9 (27.5)	55.8 (30.1)	58.0 (30.8)	56.3 (32.6)	56.7 (30.9)	0.195	0.034
Grip	67.2 (23.0)	56.2 (30.4)	56.5 (29.9)	58.4 (27.7)	57.0 (29.1)	0.232	0.039
Tactile Sensitivity	65.3 (26.9)	58.3 (30.1)	55.1 (29.4)	62.5 (29.5)	58.6 (29.5)	0.330	0.156
Skin irritation (lack of)	78.8 (18.2)	72.6 (22.0)	67.4 (28.8)	72.1 (28.4)	70.8 (26.3)	0.355	0.147
Strength	73.6 (21.0)	61.9 (25.4)	53.4 (30.7)	58.0 (30.2)	57.9 (28.7)	0.004	<0.001

* Where 0 = very bad, 100 = very good.

## Discussion

Surgical gloves are the critical barrier between the surgical team and the patient within the sterile field. Our study is the first of its kind in that we systematically compared the durability and acceptability of surgical glove types in a randomized clinical trial conducted in a real-world environment. Clinical trials conducted in real-world settings are logistically challenging because of competing clinical priorities.^16,17^ Study team members fit and re-fit participants with gloves to ensure acceptability to providers for the specific clinical tasks required in each case. The randomization and 10-period rotation allowed for scheduled, repeated exposures of each study group to each of the study gloves. In this design, each of the study participants and/or surgical groups served as their own controls.

Glove perforation rates reported in the literature are highly variable^2–8,18,19^ consistent with the diversity of surgical cases, glove types, specialty instruments or devices,^19^ provider types,^21^ and resource settings^3^ reflected in the published literature. Our overall failure rates ranged from 7.7% to 11.1% for undergloves and 17.5%–23.7% for overgloves, which is squarely in the middle of reported ranges of 7%–60%.^2–8,18–21^

Surgical team members only detect a minority of perforations in gloves during operative procedures.^13,14,21^ While the risk to provider and patient due to these breaches in maximum sterile technique has yet to be quantified in terms of clinical outcomes, there is evidence that bacteria from the provider’s hands can enter the sterile field via perforated gloves.^13^ The ability of providers to identify gloves whose barrier function has been compromised is important in the maintenance of the sterile field, particularly during technically difficult, prolonged, or high-risk (for SSI) cases. In our study, participants wearing Brand 1 gloves were more likely to be able to detect perforations intraoperatively and proactively change gloves during the procedure. The ability of surgical team members to detect intraoperative failures should be considered when evaluating gloves for a facility.

Surgical case factors that may elevate risks include long durations,^21^ specific procedures, or instruments that are associated with higher perforation rates.^20^ In the adjusted analysis that accounted for the duration, surgical service type, and provider type, the differences in failure rates between brands were the same. Despite known associations between surgery type and length on perforation rates, there are no standard recommendations for the frequency of glove changes that should occur in most operative procedures. Given the frequency of undetected glove perforations, scheduled glove changes could limit the amount of time a provider is using a compromised glove.

Limitations of this study include that it was conducted at a single site, and providers were allowed to opt out of participation if the surgical case required a specific, non-study glove type. In addition, providers chose from a range of glove options within each brand to ensure an appropriate glove type for the procedure was available for each clinical case. There were similar glove types available for each brand in an attempt to maintain consistent glove type choices across the 4 brands, but certainly, the choices necessary to perform this real-world study in a clinical environment could have introduced bias. Study questionnaires were limited potentially by a failure of participants to recall specific features of study gloves as they were used in a subset of the providers’ cases for any given period.

In this study, we tested over 7,000 gloves using trained technicians and validated WLT methodologies, over a 2-year period, involving multiple surgical services and hundreds of surgical procedures. The data collected allows for a comprehensive and updated view of the risks of glove failures in modern surgery. In the era of double-gloving as a standard for surgical team members, our high perforation rates raise the question: Is double-gloving enough? Protocols already exist in which additional glove changes are performed, in efforts to prevent contamination of the wound and prevent SSIs.^22^ Preservation of glove integrity at the surgeon/patient interface is imperative to maintain maximum sterility and should be prioritized in both glove design and gloving procedures in the operating room.

## Data Availability

The data can be shared for research purposes upon request by contacting the Principal Investigator Michelle Doll, michelle.doll@vcuhealth.org, but may require additional permissions from the institutional review board and the original data holders.

## References

[1] Albin MS , Bunegin L , Duke ES , Ritter RR , Page CP. Anatomy of a defective barrier: sequential glove leak detection in a surgical and dental environment. Crit Care Med 1992;20:170–184. 1737454

[2] Tanner J , Parkinson H. Double gloving to reduce surgical cross-infection. Cochrane Database Syst Rev 2006;2006:CD003087. doi:10.1002/14651858.CD003087.pub2 PMC7173754 16855997

[3] Bekele A , Makonnen N , Tesfaye L , Taye M. Incidence and patterns of surgical glove perforations: experience from Addis Ababa, Ethiopia. BMC Surg 2017;17:26. doi:10.1186/s12893-017-0228-8 28320370 PMC5359816

[4] Goldman AH , Haug E , Owen JR , Wayne JS , Golladay GJ. high risk of surgical glove perforation from surgical rotatory instruments. Clin Orthop Relat Res 2016;474:2513–2517. doi:10.1007/s11999-016-4948-3 27339122 PMC5052191

[5] Tlili MA , Belgacem A , Sridi H , et al. Evaluation of surgical glove integrity and factors associated with glove defect. Am J Infect Control 2018;46:30–33. doi:10.1016/j.ajic.2017.07.016 28893444

[6] Muto CA , Sistrom MG , Strain BA , Farr BM. Glove leakage rates as a function of latex content and brand: caveat emptor. Arch Surg 2000;135:982–985. doi:10.1001/archsurg.135.8.982 10922263

[7] Sayin S , Yilmaz E , Baydur H. Rate of Glove Perforation in Open Abdominal Surgery and the Associated Risk Factors. Surg Infect (Larchmt) 2019;20:286–291. doi:10.1089/sur.2018.229 30735109

[8] Partecke LI , Goerdt A-M , Langner I , et al. Incidence of microperforation for surgical gloves depends on duration of wear. Infect Control Hosp Epidemiol 2009;30:409–414. doi:10.1086/597062 19335225

[9] Geelhoed GW. Hand in glove. Crit Care Med 1992;20:159. doi: 10.1097/00003246-199202000-00002. 1737452

[10] Kampf G , Kramer A. Epidemiologic background of hand hygiene and evaluation of the most important agents for scrubs and rubs. Clin Microbiol Rev 2004;17:863–93. doi: 10.1128/CMR.17.4.863-893.2004. 15489352 PMC523567

[11] Critchley E , Pemberton MN. Latex and synthetic rubber glove usage in UK general dental practice: changing trends. Heliyon 2020;6:e03889. doi: 10.1016/j.heliyon.2020.e03889. 32405551 PMC7210590

[12] Marie-Noëlle Crepy . Rubber: new allergens and preventive measures. Eur J Dermatol 2016;26:523–530. doi:10.1684/ejd.2016.2839 28007673

[13] Harnoss JC , Partecke LI , Heidecke CD , Hübner NO , Kramer A , Assadian O. Concentration of bacteria passing through puncture holes in surgical gloves. Am J Infect Control 2010;38:154–8. doi: 10.1016/j.ajic.2009.06.013. 19822380

[14] Meakin LB , Gilman OP , Parsons KJ , Burton NJ , Langley-Hobbs SJ. Colored indicator undergloves increase the detection of glove perforations by surgeons during small animal orthopedic surgery: a randomized controlled trial. Vet Surg 2016;45:709–714. doi:10.1111/vsu.12519 27412490 PMC4973670

[15] ASTM Standard D5151, 2023, “Standard Test Method for Detection of Holes in Medical Gloves,” West Conshohocken, PA; ASTM International: 2023, DOI: 10.1520/D5151-19.

[16] McCulloch P , Taylor I , Sasako M , Lovett B , Griffin D. Randomised trials in surgery: problems and possible solutions. BMJ 2002;324:1448–51. doi: 10.1136/bmj.324.7351.1448. 12065273 PMC1123389

[17] Kao LS , Aaron BC , Dellinger EP. Trials and tribulations: current challenges in conducting clinical trials. Arch Surg 2003;138:59–62. doi:10.1001/archsurg.138.1.59 12511152

[18] Misteli H , Weber WP , Reck S , Rosenthal R , Zwahlen M , Fueglistaler P , Bolli MK , Oertli D , Widmer AF , Marti WR. Surgical glove perforation and the risk of surgical site infection. Arch Surg 2009;144:553–8. doi: 10.1001/archsurg.2009.60. 19528389

[19] Zhang A , Gao X , Ruan X , Zheng B. Effectiveness of double-gloving method on prevention of surgical glove perforations and blood contamination: A systematic review and meta-analysis. J Adv Nurs 2021;77:3630–3643. doi: 10.1111/jan.14824. 33733484

[20] Goldman AH , Haug E , Owen JR , Wayne JS , Golladay GJ. High risk of surgical glove perforation from surgical rotatory instruments. Clin Orthop Relat Res 2016;474:2513–2517. doi:10.1007/s11999-016-4948-3. 27339122 PMC5052191

[21] Thomson I , Krysa N , McGuire A , Mann S. Recognition of intraoperative surgical glove perforation: a comparison by surgical role and level of training. Can J Surg. 2022;65:E82–E88. doi: 10.1503/cjs.016720. 35135784 PMC8834241

[22] Albert H , Bataller W , Masroor N , Doll M , Cooper K , Spencer P , Winborne D , Zierden EM , Stevens MP , Scott M , Bearman G. Infection prevention and enhanced recovery after surgery: A partnership for implementation of an evidence-based bundle to reduce colorectal surgical site infections. Am J Infect Control 2019;47:718–719. doi: 10.1016/j.ajic.2018.11.004. 30584020

